# PW03-008 – Mitochondrial disturbances in Schnitzler syndrome

**DOI:** 10.1186/1546-0096-11-S1-A234

**Published:** 2013-11-08

**Authors:** A Sediva, H Hansikova, J Sladkova, M Rodinova, Z Hajkova, J Zeman, P Szturz

**Affiliations:** 1 Department of Immunology, Motol University Hospital, 2nd School of Medicine, Charles University, Prague, Czech Republic; 2 Department of Pediatrics, First Faculty of Medicine, Charles University and General University Hospital, Prague, Czech Republic; 3 Clinic of Hematooncology, Faculty of Medicine, Masaryk University and University Hospital, Brno, Czech Republic

## Introduction

Schnitzler syndrome is an autoinflammatory disorder of unknown etiology. At least some of its clinical presentation is mediated through an activation of inflammasome and release of IL-1, as was repeatedly demonstrated by a prominent therapeutic effect of Il-1 blockade.

## Objectives

Recent reports bring an evidence of an important role of mitochondria in inflammasome activation and in a pathogenesis of autoinflammatory diseases. We have therefore investigated mitochondrial function and structure in patients with Schnitzler syndrome.

## Methods

Activity and amount of oxidative phosphorylation complexes (OXPHOS) were analysed by spectrophotometry, histochemistry and imunoelectrophoretic methods in fibroblast cell lines derived from skin biopsies of three adult male patients with Schnitzler syndrome. Ultrastructure of mitochondria, mitochondrial network and reactive oxygen species (ROS) were analysed by fluorescent and electron microscopy.

## Results

The activities and amount of OXPHOS complexes I, III and IV were decreased in patients with Schnitzler syndrome. Interindividual differences in the degree of impairment (from severe to moderate) in analyzed mitochondrial parameters were found. Content of ROS, previously suggested as main inducers of inflammasome, were not significantly increased in cells with Schnitzler syndrome. We, however, did find consistent and prominent changes in mitochondrial structure of all three patients. Disturbed mitochondrial network and mainly abnormal, partially swelling mitochondria with unusual and sparse cristae were characteristic for all patients. We did further notice marked accumulation of neutral lipids in all tested fibroblasts.

## Conclusion

Severe structural damage of mitochondria associated with milder functional changes represented a consistent feature found in all tested Schnitzler syndrome patients. *Supported by RVO-VFN64165/2012*

## Disclosure of interest

None declared

